# Examination of adolescent and youth modern contraceptive users’ perceptions on how religion influences contraceptive use and their rationale and circumstances of use: qualitative evidence from Burkina Faso, Kenya and Niger

**DOI:** 10.1007/s12546-025-09362-5

**Published:** 2025-01-17

**Authors:** Ilene S. Speizer, Fiacre Bazie, Amelia Maytan-Joneydi, John A. Mushomi, Sanoussi Chaibou, Kindo Boukary, Balki Ibrahim Agali, Julius Rwenyo

**Affiliations:** 1 Department of Maternal and Child Health and Carolina Population Center, University of North Carolina at Chapel Hill, Chapel Hill, NC USA; 2 Institut Supérieur des Sciences de la Population (ISSP) at the Université Joseph Ki-Zerbo, Ouagadougou, Burkina Faso; 3 Carolina Population Center, University of North Carolina at Chapel Hill, Chapel Hill, NC USA; 4 African Institute for Development Policy (AFIDEP), Lilongwe, Malawi; 5 Department of Population Studies, Makerere University, Kampala, Uganda; 6 Groupe de Recherche et d’Action Pour le Développement (GRADE Africa), Niamey, Niger; 7 Groupe de Recherche et d’Action Pour le Développement (GRADE Africa), Zinder, Niger; 8 African Institute for Development Policy (AFIDEP), Nairobi, Kenya

**Keywords:** Religion, Adolescent, Youth, Contraceptive use, Burkina Faso, Kenya, Niger

## Abstract

While religion is a key determining factor of contraceptive use, few studies examine how religion influences adolescent and youth contraceptive attitudes, beliefs, and use. We use recently collected (August–November 2022) qualitative data from Burkina Faso, Kenya, and Niger among young users of modern contraception who practice Christianity or Islam. In-depth interviews with married and unmarried young women ages 18–24 years were conducted in two sites in each country to obtain a mix of religions and method users. In each country, many young Christian and Muslim women perceived that their religion is not supportive of contraceptive use. Some nuances around perceived acceptability of use were identified in Niger and among Muslim women in Kenya particularly for married women for spacing or health reasons. Reasons given for using related to realities of life, personal choices, and that use is their prerogative and God will forgive them. Most married women felt there would be few consequences if their religious community learned of their use whereas unmarried young women feared more consequences from their religious and broader community. These findings demonstrate that while religion is important in all three study contexts, decisions around contraceptive use among the young women included were not necessarily influenced by their religious beliefs and practices. As a greater number of young people adopt contraception, with or without perceived religious support, social norms are likely to change leading to increased access to contraception for all young women, married and unmarried, when or if they need it.

## Introduction

Contraceptive use provides numerous health and well-being benefits to families through the spacing of births, avoidance of unintended or unwanted pregnancies, and delay of childbearing onset to complete education or economic opportunities (Cleland et al., 2012). For young people, recent studies have demonstrated the advantages of avoiding rapid, repeat pregnancies and the opportunities created by remaining in school and delaying marriage and childbearing (Candra-Mouli et al., 2017; Ganchimeg et al., 2014; Norton et al., 2017). Contraceptive use among married and unmarried, sexually active young people helps to meet these important health and well-being outcomes. Identifying and addressing adolescent and youth sexual and reproductive health needs is an increasingly important global initiative. For example, Sustainable Development Goal 3.7 seeks to attain by 2030 universal access to sexual and reproductive health care services, including family planning (FP); this includes access for adolescents (ages 10–19) and youth (ages 20–24). Further, the FP2030 initiative promotes the right of all people to decide freely whether, when, and how many children to have and supports country-led commitments to FP programming and increasingly there is a focus in these country commitments on supporting adolescent and youth access to and use of contraception.

Global estimates demonstrate increases in contraceptive use from 1970–2019 in all age groups, including among the youngest age groups of 15–19 and 20–24; however, increases among the youngest groups have not been as large as those of their older counterparts (Haakenstad et al., 2022). Further, young contraceptive users, especially those who are not in union, typically use pills and condoms which are user dependent methods and have lower effectiveness rates than longer acting methods such as injections and implants (Haakenstad et al., 2022; Bertrand et al., 2023), increasing their risk of an unintended pregnancy. When asked about their reasons for choosing these methods, young people often report concerns about side effects and the importance of rapid return to fertility as their explanations (Calhoun et al., 2022). Additionally, unmarried young people often seek out methods that are easily accessible including condoms and emergency contraception which do not require visiting a facility to obtain. Given the strong social sanctions against premarital sex in many contexts, including sub-Saharan Africa where this study takes place, sexually active young people often lack easy access to more effective, longer-acting methods.

Recent quantitative studies that examine contraceptive use and unmet need among adolescents and youth in low- and middle-income countries demonstrate factors at the individual and community-levels of the socioecological model (McLeroy et al., 1988) associated with use and non-use. A common finding in these studies is that Muslim adolescents and youth are less likely to use contraception than their Christian (Catholic or Protestant) counterparts (Akinkorah, 2020; Tigabu et al., 2018; Obasohan, 2015). A systematic review on factors affecting sexual and reproductive health of Muslim women of all ages that included both quantitative and qualitative studies demonstrated that many of the Muslim women had poor knowledge and negative attitudes toward sexual and reproductive health (SRH); this affects their access to and use of SRH services, including FP services (Alomair et al., 2020). Further, in a recent study from Nigeria, Adedini et al. (2018) find that women exposed to favorable family planning messages from religious leaders were significantly more likely to be currently using contraception than their counterparts who were not exposed to religious leaders talking about family planning, controlling for many demographic factors. This suggests that while religion may be a detractor of contraceptive use, religious leaders and their voices can also be supportive of use.

Qualitative studies across a number of locations and target groups including women, men, providers, and religious leaders have demonstrated varied perspectives on religion and religious teachings and contraceptive use across contexts. These studies have demonstrated high knowledge of family planning methods among religious leaders (Aristide et al., 2020; Barro & Bado, 2021; Barro et al., 2021); however, there are varying perspectives on the acceptability of use within and across religions (Abdi et al., 2020; Ataullahjan et al., 2019; Bado et al., 2020; Barro & Bado, 2021; Chalem et al., 2023; Egeh et al., 2019; Kenny et al., 2021). Further, women who are included in qualitative studies also report varying perspectives on whether FP is acceptable in their religion and diverse reasons for using a method, even in contexts where they feel methods are not permitted (Alomair et al., 2020; Aristide et al., 2020; Ataullahjan et al., 2019; Sahu & Hutter, 2012; Sundararajan et al., 2019). Finally, only one recent study was found that included the perspectives of young people and determined the role of their perceptions about religion on their sexual and reproductive health behaviors; this study was from Niger (PSI, 2017). This study demonstrated that while religion is important to the young people, the primary determining factor for use among the married women was the husband’s support. Generally, it was found that while the youth were religious, they did not report that religious leaders were influential on matters related to sexual and reproductive health including family planning. Among the many qualitative studies found, only the one from Niger explicitly focused on the perspectives of young people and that study did not necessarily include adolescent or youth users to understand their perspectives on how religion affects their use.

The objectives of this study were to fill a gap in our understanding of the role of religion and religious leaders on female adolescent and youth modern contraceptive use in Burkina Faso, Kenya, and Niger. To do this, we present findings from a recent qualitative study that focused on understanding young women modern contraceptive users’ a) perceptions of their religion’s acceptability of contraceptive use; b) rationale for contraceptive use; and c) perceived consequences on non-sanctioned use.

## Methods

### Study contexts

This qualitative study was undertaken in three countries: Burkina Faso, Kenya, and Niger. In each country, two mostly urban study sites were selected with the objective of obtaining data that are comparable in terms of religious affiliations across the sites. First, Burkina Faso is a diverse country with a mix of religions. The most recent 2021 Demographic and Health Survey revealed that 63.4 percent of the population of women surveyed were Muslim, 24.7 percent were Catholic, and 8.1 percent Protestant; the remaining 3.7 percent of the women practiced a traditional religion or had no religion (INSD and DHS Program, 2023). This religious distribution is similar in the two study sites from Burkina Faso: Ouagadougou (capital) and Bobo-Dioulasso. In Kenya, the second study country, we included two sites: Mombasa that has about a third of the population that is Muslim and two-thirds that are Christian, including Catholic, Protestant, and Evangelical; and Wajir that is 97 percent Muslim. Finally, we included Niger where about 99 percent of the population is Muslim; in Niger, we included Niamey (capital) and Zinder, a more conservative city.

### Study sample and recruitment

In each study country, the study teams undertook in-depth interviews with young women (ages 18–24^1^). Because of the focus on including young modern contraceptive users, it was decided to focus on this slightly older age group since across the three countries the most recent Demographic and Health Survey demonstrated that the percentage of women ages 15–17 years using modern contraception was low (5.2% in Kenya, 2022; 5.9% in Niger, 2012; and 6.8% in Burkina Faso, 2021). Eligibility criteria for the young women, the focus of this paper, was that they had to be currently using a modern method of contraception (i.e., IUD, implant, injectable, pills, condoms) and had to be practicing their Muslim, Catholic, or Protestant religion. We defined practicing the religion here as attending weekly church services and being involved in activities within their religious community outside of church services among the Catholic and Protestant participants and for the Muslim participants, praying five-times a day and being involved in activities at the mosque or with their religious community. These objective measures of practice were meant to be proxies for capturing religiosity. The decision to include users who were practicing their religion was based on our interest in understanding how these young women navigate their use in varying supportive or unsupportive contexts. Non-users were not included since they have many reasons for non-use, possibly including religious restrictions but also related to non-need (i.e., a desire to get pregnant), and other individual or supply side factors.

A minimum of 12 interviews with young women per study site in each country were required with ideally a mix of religions, if applicable, and marital statuses. In Niger, additional interviews were undertaken to supplement the 12 required interviews per site. Recruitment of participating young women was undertaken slightly differently in each country. Generally, the study teams were meant to work through community health workers to help identify known contraceptive users who practiced the different religions expected from each site. The community health workers worked closely with the trained female interviewers to identify potential candidates for in-depth interviews. Once identified, the trained interviewer met one-on-one with the young person and asked the relevant contraceptive use and religious practice screening questions to determine if the young person was indeed eligible. All eligible young women were asked for informed consent to participate. Data collection happened between August and November 2022.

As seen in Table 1, in Burkina Faso the study team was able to recruit a diverse pool of women who were spread across the three religions (Muslim, Catholic, and Protestant) and marital statuses (unmarried and married). In Kenya, participants were typically older and mostly married in both sites and in Wajir, all participants were Muslim. In Kenya, the team did not distinguish whether the Christian participants were Catholic or Protestant, although sometimes this is clear from the responses given. In Niger, participants were older and included Muslim and some Christian (mostly Protestant) participants; several unmarried participants were included in Niger. Also shown in Table 1 is the current modern method used by young women in the sample. Differences are seen between cities (within a country) and across countries. The methods used may reflect these young women’s access to the different methods at facilities as well as socially accepted methods within the varying cities, religions, and cultural contexts.

**Table 1:** Number of female adolescent and youth participants by country and city and demographic characteristics, 2022

Characteristic	Burkina Faso		Kenya		Niger	
	Bobo Dioulasso	Ouagadougou	Mombasa	Wajir	Niamey	Zinder
*Age*						
18–20	1	3	3	0	2	1
21–22	2	1	2	1	1	4
23–24	9	8	7	10	15	9
25–26	0	0	0	1	1	2
*Religion*						
Catholic	4	4			1	1
Protestant	4	4			7	3
Christian*			9	0		
Muslim	4	4	3	12	11	12
*Marital status*						
Unmarried	6	6	3	0	6	7
Ever married	6	6	9	12	13	9
*Current method*						
IUD	1	0	1	1	0	1
Implant	6	2	5	4	6	2
Injectable	2	4	5	2	3	3
Pill	3	2	0	5	10	7
Condom	0	4	0	0	0	3
No method			1			

^*^In Kenya, Christian women were not distinguished by whether they were Catholic or Protestant, although some discussed a specific denomination during the interview. In addition, in Kenya, one young woman interviewed did not report current use of a method; in both Bobo Dioulasso and Niamey, one pill user also reported condom use

### Data collection methods

Study teams across the three sites jointly developed a semi-structured interview guide that asked questions to the young women about their contraceptive experience, decision-making around contraceptive use, perceptions of their religion’s acceptance of contraception, and perceived consequences of their contraceptive use. The interview guide was translated into multiple local languages to be used in each study context. All recruitment materials, consent forms, and interview guides were approved by the in-country ethics review panels including for Burkina Faso: the Comité d’Ethique pour la Recherche en Santé in Burkina Faso (#2022–06–122); for Kenya: the AMREF Ethical Review Committee, (ESRC P 1299–202), and the National Commission for Science, Technology, and Innovation (NACOSTI) in Nairobi (NACOSTI/P/22/19360); and for Niger the Comité Nationale d’Ethique pour la Recherche en Santé (#41/2022/CNERS). The protocol was also approved by the Institutional Review Board at the University of North Carolina at Chapel Hill (#22–1125).

In all countries, the interviews were undertaken by a trained female interviewer and in a location that was private and comfortable to the participating young person. Prior to the interview, the interviewer read the consent form to the young woman in the local language and asked for written consent to participate. All interviews were recorded, transcribed, and translated from the local language into French (Burkina Faso and Niger) or English (Kenya) for analysis. Data were uploaded into Dedoose, a qualitative data software that is useful for collaborative coding and analysis.

### Analysis methods

For each country, a team of 4–5 people were involved in coding; at least one person was involved in coding for all three countries and two others coded for both Niger and Burkina Faso; this permitted the possibility for supporting cross-country codes and understanding. Given the timelines for data collection (July 2022 in Kenya; June-July 2022 in Burkina Faso; and September–October 2022 in Niger), coding happened sequentially for the countries with Kenya happening first, followed by Burkina Faso, and then Niger. As a first step, we developed an initial codebook based on the interview guides and in each country all coders coded the same interview. Following this initial coding, the team met to review coding decisions and to resolve differences and identify additional codes necessary to capture the nuance of the study context. Once this initial process was complete, the team coded a second interview and did another round of comparison to ensure alignment across the team’s code application and to ensure inclusion of a full set of relevant codes. After the second interview was jointly compared and agreement was established, each coder was responsible for coding interviews; each interview was coded by one person and the initial two interviews were recoded. Once all data were coded for each country, a thematic analysis was undertaken to identify important themes within and across the countries. For each country, we undertook a deep analysis of the adolescent interviews; the country-level findings are presented elsewhere. In this paper, we undertake a cross-country examination to better understand similarities and differences across the study contexts. Below we present the results by the identified themes. For quotes that come from the Niger and Burkina Faso interviews, the quotes were translated into English and then colleagues from the specific countries ensured that the meaning remained in the final English versions included.

## Results

Our analysis of these data led to three high-level themes that we describe below. The themes we cover are a) perspectives on religion’s acceptability of contraceptive use; b) young women’s reasons for current contraceptive use; and c) perceived consequences of unsanctioned contraceptive use. The analysis presented below demonstrates similarities and differences observed across the three study contexts as well as by religion.

### Perspectives on religion’s acceptability of contraceptive use

In this section, we first outline the more common perspective that religion does not support contraceptive use; this perspective was found across the three study countries, religions, and marital statuses. Then we highlight the scenarios where the young women believed that their religion did support use, particularly for spacing and the health of the mother or child; this was more commonly reported among women of all religions in Niger and by some Muslim women in Kenya. Finally, we present the perspectives on non-marital sex and non-marital contraceptive use that were universally unsupportive across the countries and religions.

The young women reported that they learned about these religious perspectives in public fora such as during sermons or in more subtle ways as part of activities within their religious community. Further, discussions with friends and families were another way that these young women learned about the perspectives around contraceptive use within their religious community. Much of the discussion about perspectives toward family planning use was about any use with less focus on the religion’s acceptability of specific methods.

### Religion not supportive of contraceptive use

In Burkina Faso, the general sentiment among Muslim, Catholic, and Protestant young women was that their religion did not support contraceptive use. This was related to the fact that their religions encourage them to have the number of children that God has in mind for them. For example, this married young Muslim woman from Ouagadougou says:

Most Muslims say it's forbidden for a young woman to use contraception. They say it's written in the Quran that family planning is not allowed in Islam. According to the Muslim religion, every woman must bear the number of children God has given her and not try to limit it by using contraception, otherwise it's a sin.

Another young Muslim married woman from Bobo Dioulasso says that she learned about these negative perspectives around contraceptive use from her religious teacher:

Interviewer: But when you say it's not allowed, where did you hear that?

Respondent: Our Quranic teacher tells us about it; often in these readings he says that a married woman must not use methods; that God condemns it. That's where I heard it.

Likewise, this Catholic married woman from Bobo Dioulasso also gives her understanding of her religion’s non acceptance of any family planning method:

Interviewer: ok, what does your religion say about FP? As far as your religion is concerned, what is doable and what is not doable for FP?

Respondent: religion is against FP

Interviewer: why?

Respondent: It's against the use of contraceptive methods; whether it's the pill or other methods, religion is against it.

In the Niger context, there were also negative sentiments found about contraceptive use from both Muslim and Christian young women. For example, some of these women talked about birth spacing being forbidden or any method use not being allowed as indicated by this married Muslim woman from Niamey:

Our religion has forbidden us to put intervals between our children. It has forbidden us to use family planning or to put anything to put an interval between our children, it has forbidden that.

And this married Catholic woman from Niamey:

Our religion categorically forbids family planning.

And this young, unmarried Protestant woman from Niger says that contraception is not allowed but then mentions that some natural or traditional methods are authorized by her religion:

Well, there's no part of the Bible to guide me to take contraceptives because religion has firmly condemned this in the Bible, it's a sin to take modern contraceptive products the Bible has its own way of doing family planning... Within the Christian religion it's only natural contraceptive methods that are allowed for example exclusive breastfeeding for two years; taking the temperature, also calculating the calendar three days before ovulation and three days after ovulation and that's it.

Similarly, many respondents from Kenya believed that their religion did not support family planning use. This was the general sentiment from the Christian women as illustrated by this married woman from Mombasa:

Interviewer: Oh okay… you have told me that you go to church and that you also do other things in church, when deciding to start using FP, did you consider religion

Respondent: Mmmm… now, I think I went against religion because it does not allow.

Further, this married Muslim woman from Kenya reports her understanding of disadvantages with contraception which reflects what she understands from her religious leaders about contraceptive use not being acceptable:

Respondent: First of all, using FP is a sin before God because God prohibited to stop producing; then some people when they use FP, they fail to produce and others can produce babies with one leg (lame babies); so, they say FP spoil the eggs and when it reaches the time to produce it causes problems.

Interviewer: Okay, so first it is a sin, then it spoils our eggs…

Respondent: Yes, it spoils our eggs and one can get lame children.

### Cases where young women perceive religion as supportive of contraceptive use

In Niger among both Muslim and Christian young women and in Kenya among Muslim women, we heard from a number of both married and unmarried interviewees about scenarios where religion is accepting of modern contraceptive use. This was less commonly reported in Burkina Faso among women of any of the religions. Those who reported support for contraceptive use often linked it to the benefits of spacing births 2–3 years or to the importance of supporting the health of the mother or baby. In Kenya, where a number of women had cesarean sections, there was a perspective among the Muslim women that the woman should rest after this surgery. For example, this Muslim married woman from Wajir reports:

I swear it is my husband who advised me to use family planning and I had to consider it. I do not think religion is an impediment because I used it given the fact that I had gone through Caesarean section and therefore FP was key for my health. Me using was squarely for purpose of my health.

Other Muslim women from Kenya talked about using for spacing and health reasons as stated by this young married woman from Wajir:

Yes, I have asked religious leader who told me it recommended for mother to breastfeed her child for two consecutive years. So, if you’re taking for the purpose of health its permissible for that matter I have taken the pills.

Further, this forward-thinking young married Muslim woman from Wajir presents an explanation why family planning is acceptable for spacing purposes from her perspective:

…Because there are religious misconceptions among our people, they tend to think that using family planning is to denounce the religion. The same way Allah has granted us technological advancements such as use of phones is the same way that Allah has granted us family planning in order to allow us space children, and it is because of this reason that I belief the religion has no ill will against family planning.

In Niger, many Muslim and some Christian women reported that their religion was supportive of contraceptive use for spacing purposes. For example, this unmarried Muslim woman from Zinder who talks about the acceptability of contraceptive use while a woman is breastfeeding:

Well, that's what our religion has allowed concerning family planning: there are women who give birth every year, as soon as she gives birth 40 days later she can become pregnant if she happens to have intercourse. For these types of women, religion allows them to use a contraceptive method until her child is weaned, but after weaning her child to stop the contraceptive method and continue giving birth.

In addition, the use of contraception for spacing was reported to be widely discussed through the mass media and through religious schools as mentioned by this married Muslim woman from Zinder:

On the radio, on TV with religious programs, or at Quranic schools when people ask questions about FP, we say that it's allowed, if it's to organize your births and not to stop for good, i.e. if it's just for birth planning, it's authorized.

There were also some Christian women from Niger who provided the perspective of family planning being acceptable for spacing births. For example, this married Protestant woman from Niamey who reports that family planning is useful for ensuring that parents can take care of the children that they have:

Further clarification is that religion accepts it to plan births, to avoid children in such disarray that parents are unable to educate them or take care of the gift God has given them.

### Perspectives on non-marital sex and contraceptive use

Importantly, across the religions, countries, and marital status categories, it was consistently reported that unmarried young women should not be having sex and therefore, should not be using contraception. This was reported by married as well as unmarried women. For example, this married woman from Wajir provides her perspective on the position around sex and contraceptive use among unmarried women:

To my opinion if you’re not married and you use FP then it’s wrong because there no reason as to why you use family planning, why didn’t you guard your virginity but if you married and your husband has given you the permission then its fine.

Of note, a small number of women acknowledged that in the present day, there are young, unmarried women who are having sex and thus while the religion might not accept contraceptive use among this group, contraceptive use is better (and safer) than unprotected sex.

For example, this unmarried Muslim woman from Zinder, Niger illustrates concerns related to sexually transmitted diseases:

No method is accepted for unmarried young women, since religious leaders always say in their preaching not to practice fornication, but nowadays with all the diseases, you have to protect yourself when sleeping with a man who isn't your husband and for some young women, regretfully it’s just to be able to support themselves.

Similar sentiments were given from women of the other religions and from the other countries. For example, this Catholic married woman from Ouagadougou while saying that contraceptive use is not accepted in her religion is also acknowledging that unmarried young women have family planning needs and there are significant barriers to accessing a method.

Interviewer: What about young single women?

Respondent: In our religion, it's said in church that it's forbidden for a young, single woman to have intimate relations outside marriage. But nowadays it's difficult because young people are sexually active, it's difficult especially with boyfriends around. And since the church doesn't advise married women to use family planning, it's even harder for young unmarried women.

### Young women’s reasons for current contraceptive use

Among the young women who were married and unmarried and using contraception, we found a number of important reasons given for why they used a method, even though, in many cases, they felt it went against their religious doctrine. These reasons fell into three main categories: realities of life; personal choice and responsibility; and God will forgive.

### Realities of life

Many women talked about how life these days is complicated and spacing births or delaying non-marital births is helpful for them to meet their personal and family goals. This included being able to afford schooling for the children they already have, as well as any future children. In addition, they discussed wanting to complete their own schooling and that the costs of living are higher than they were when their parents were having children. All of these issues led them to accept family planning as a solution to succeed, even though it may not be considered acceptable in their religion.

This married Christian woman from Kenya provides clarity on many of these issues:

Yes, those in marriage should take care of themselves and plan because with the current economic situation, there is no alternative but to plan. They have to (…) life has become difficult and we cannot just follow religion and say we will just give birth and give birth… we have to plan ourselves.

Further, this married Muslim woman from Niger talks about the costs required to ensure the health and well-being of her children and herself as reasons why she needs to use contraception:

Even if religion doesn't allow it, there's your health and living conditions. There aren't enough resources to bring up a lot of children, and you can't combine studying with having a lot of children. That's all I can say, and if I have to give birth every year, all these things will force me to use FP.

The unmarried women also gave similar reasons for using family planning against their religious guidance; these reasons related to economic considerations, education, and life challenges. This unmarried Muslim woman from Burkina Faso provides a clear perspective on this:

Interviewer: you said you do FP, even if religion is against it would you be willing to continue using it?

Respondent: every person does things for reasons if I do it it's because I have a plan and needs, I'll stop once the need is met.

Interviewer: What can motivate a human being to use contraception, what are the points of view?

Respondent: If you don't have money, a job or a husband, it's complicated to have children, so if all these conditions come together, I think I can stop and have children.

### Personal choice and responsibility

Many young women across the countries reported that it was important for them to use contraception because rearing children is their responsibility and if they have (too many) children they may not be able to care for them. This explanation of personal choice and responsibility overlapped with the explanations around realities of life but also leaned toward responses that were more autonomous in nature. This included married women who felt that having children too closely spaced together would affect their and their children’s health.

This young, married Muslim woman from Ouagadougou, when asked about what is not allowed in her religion, makes the point that women need to do what is right for them:

Ah, well, it's difficult for me to know, as long as they've said that it's God who has said that the Muslim religion forbids family planning, I think we should take it that way and not try to understand too much. But on an individual basis, I think everyone should do what suits them, even if religion doesn't allow it. You don't need to talk to anyone about what you're doing; it's your decision, and as a married woman you have to do what you think is right and, above all, what's right for you.

Earlier in the interview, this same woman from Burkina Faso again emphasizes the personal choice around contraceptive use in the current environment.

Nowadays, the decision to use FP is up to each and every one of us, even if it's not allowed in Islam.

A young married Muslim woman from Kenya also provides this perspective of contraceptive use being a personal matter when she was asked about how her religious community can support young women’s family planning use:

Respondent: I think on this matter the community doesn’t have to accept because someone decides at a personal level what to do with their body, whether to put family planning or not so they should not bring in whether the community or religion will accept or not.

Interviewer: So in your opinion religion should not interfere?

Respondent: Yes it’s not supposed, it shouldn’t say whether it is right or wrong because it is a personal decision, yeah.

Unmarried women also felt it was their responsibility to avoid unwanted pregnancies for their own futures but also for their relationships with their families that could be affected if they had children out of wedlock. In some cases, unmarried women were supported by their mothers to use family planning with the goal of avoiding the risk of a non-marital birth. For example, this unmarried Protestant woman from Niger states her reasons for not consulting anyone before using family planning, indicating that this was her personal choice and saves her family from potential scandal:

Interviewer: My dear, did you take religion into account when you decided to use FP?

Respondent: If I had to take religion into account, I wasn't going to use FP because in religion, I mean in the Christian religion, a child is a blessing and family planning allows you to terminate a pregnancy or forbid access to a pregnancy. So when I made my decision, I took it personally to save my image and the image of the family.

### God will forgive

A number of the young women interviewed across the countries acknowledged that they were using family planning even though they thought this went against their religious doctrines. They often explained that it was important for them to use for the reasons listed above including their own and their children and families’ health and wellbeing, the cost of living and educating children, and the difficulties in the current environment. Given these realities of life, the young women often said that God will have to forgive them or that God, and not their religious community (or family), will be their judge.

Some examples of this perspective from Muslim women who more often gave this reasoning are given below. For example, this married Muslim woman from Ouagadougou, after saying that her husband told her that the Quran does not permit contraceptive use, was asked how this information affected her decision to use:

Sincerely, when he [her husband] told me about the prohibition of FP in our religion, I really wasn't happy and I asked God to forgive me, because it's not easy for us women either. If it's about children, we want to have them as religion dictates, but we also need to know how to limit and space our births so we can take good care of them. So it was with a view to taking good care of my children that I decided on my own to use contraception.

And this married Muslim woman from Wajir who explains that even though she knew her religion is against her use, she will repent to God:

Yes, before I gave birth I used to hear that family planning is not good from other people and it was not based on religious sermons to inform people. So you just hear about negative aspect but because of circumstances I have to use. Religion wise I know it’s not good and I will repent to God.

Unmarried women also gave similar perspectives of the importance of using against religious doctrines and that the judge will be God. This unmarried Muslim woman from Burkina Faso, when asked how her contraceptive use will impact her religious beliefs, sums up her need to use, and how God will have to forgive her:

(Silence) Despite the fact that I used the method, I continue to practice my religion without any problem. Often you do things out of obligation because you have no choice; it's the same thing by the way, we know that religion has forbidden the use of contraception especially among young unmarried girls, it's because I have no choice, I did it asking God's forgiveness because it's to protect myself because I have no choice.

### Perceived consequences of unsanctioned contraceptive use

For the most part, unsanctioned use across the religions did not seem to come with concerns about significant consequences, particularly for the married women. For example, several married women said that if their husband was on board with their use, the religious leaders and community could not push back. In addition, those who talked about potential perceived consequences said that they expected that their religious leaders might just counsel or advise them on their use but would not forbid their use. Further, many women said that use is a discrete matter and there is no reason that their religious leaders or religious communities will find out about their use. Finally, there were some of the participants that acknowledged that times are changing, and people are becoming somewhat more open minded about contraceptive use and thus it is becoming more accepted, even if religious leaders feel the need to counsel against use with the young women. Not surprisingly, the perspectives of the unmarried users were different, with these young women reporting that they expected more severe consequences.

This married Muslim woman from Mombasa summarizes the various issues raised about perceived consequences or lack of them:

(Sighs) There everyone has their own life, they can’t come and plan and tell me, maybe they’ll give me advise and tell me it’s not right so that if I’m able to go back I go back but they can’t force me to stop, yes. Because currently using family planning not everyone will know I have put family planning, not everyone.

And this married Protestant woman from Niamey when asked about consequences of her religious community finding out about her use reports that she expects that her and her husband will be counseled about their use and the pastor might use this as a reason to preach more against family planning use:

I think that religious leaders can call on me and my husband to ask why you did this, why you did that, even though it's forbidden by religion. Well, I don't think there will be any sanctions, but I do think that pastors can preach more to draw the attention of those who might do the same thing.

Among the unmarried young women there were two groups, those who felt that there would not be any consequences either because contraceptive use is discrete in nature so no one needs to know or because times are changing; and those who felt that there may be severe consequences if their use is detected.

For example, this young unmarried Muslim woman from Zinder reports that times have changed, and people are more open to non-marital use and that this is becoming more normative:

Interviewer: If, for example, the Muslim community or your fellow students at the Quranic school find out that you're using them, what are they going to say?

Respondent: Now people's minds are open, everyone uses these products, nowadays those who don't use them aren't many really, they're not many, if you don't use them you'll even be advised to use them.

Further, this young unmarried Catholic woman from Ouagadougou also states that generally use is a private matter and it is not possible to know if someone is using and if they are detected, there will not be any implications.

Interviewer: What consequence is reserved for single users in your religious community?

Respondent: They can't know whether you've done FP or not, how can they know that all the women there have done FP?

Interviewer: But if the religious community were to find out, what would be the consequences?

Interviewer: Even if they do, there's nothing they can do. Even if they find out, they can't say anything, and they have nothing to say.

Interviewer: Why can't they say anything?

Respondent: It's because it's not written in the Bible that its use is good or bad, so what are they going to base their words on? Even if you're questioned, you can say that it's because they say in health centers that its use is good that you're using it.

The most severe perceived consequences mentioned by the unmarried women included being publicly called out for their behaviors and being ostracized from the activities they undertake in their religious community. A few examples provided by young unmarried women in each country are provided here. First, this young unmarried Muslim woman from Zinder discusses that if her community finds out she will be branded a prostitute:

Respondent: People are going to insult me, they're going to misinterpret me.

Interviewer: What do you mean by misinterpretations?

Respondent: In the community, people will consider you a prostitute.

Further, this Protestant unmarried woman from Bobo-Dioulasso reports the consequences of a non-marital birth which are similar to the consequences of non-marital contraceptive use:

Respondent: Everyone will know that you're in this situation (that you've had sex), and they'll forbid you to do anything in the church. And when you want to get married, they won't celebrate it (your marriage) in the church, they'll celebrate it outside.

Interviewer: You've just talked about the consequences for a young girl who gets pregnant, but when they find out you're doing family planning, what would be the consequence? What punishment are they going to apply to the person?

Respondent: It's the same thing, it's not just giving birth. Even if you have sex, there's a punishment for that. If you use family planning, that means you're having sex. So it's the same punishment.

Finally, this young unmarried Christian woman from Mombasa states that the consequences will affect her social engagements and social circles:

Interviewer: What are the consequences if your religious community discovered that you used FP despite its teachings?

Respondent: The consequences?

Interviewer: Yes.

Respondent: First of all they will not want you to hang out with their daughters because they already know you are a bad influence. Secondly I can say that you won’t have friends or getting into certain circles will be like a mockery because you don’t want to get into them. You won’t participate in some activities.

## Discussion

This study is a first that examines young women modern contraceptive users’ perspectives on how their religion views contraceptive use and their rationale for use, even in circumstances where it is perceived to not be allowed by their religion. Across all sites and religions, the young women interviewed often perceived that their religion did not approve of contraceptive use. This was a particularly common perspective in Burkina Faso across the religions, sites and marital statuses and among the Christian young women in Kenya. That said, when the young women reported more flexible or accepting perspectives on contraceptive use by their religions, this was specifically for birth spacing and for the health of the mother or baby. Most notably was that these accepting perspectives were more commonly reported among both Muslim and Christian participants from Niger and by Muslim participants in Kenya. Not surprisingly, across the sites and religions, the general feeling was that contraceptive use is not accepted for unmarried young women.

The main reasons for using contraception fit into three related categories – realities of life, personal choice and responsibility, and God will forgive. Further, when asked about perceived consequences for their unsanctioned use, the married women across the countries seemed to think that the consequences would be minimal. Given the expectation of minimal consequences, these young, married women are making decisions that are best for their own and their families’ lives and are often doing this with their husband’s engagement. These women were often using to space births, post-cesarean section, and to permit their being able to support their children’s education and general well-being. This suggests that while religion is important to these women’s lives, religion is not an important influencer in contraceptive decision-making.

For the unmarried young women, their main reasons for use focused on protecting against a non-marital birth because the perceived consequences of that birth in their religious, family, and social community would be even worse than the perceived consequences of detected contraceptive use. The young unmarried women across the different country contexts are making decisions based on their current circumstances and partnerships; in these cases, they are not worried about their religion and are expecting that either their use will not be detected or that they can ask forgiveness from God. Like their married counterparts, these young unmarried users are choosing to use and view this as important to their own lives and do not think that their use will affect their religious practices and beliefs.

Our findings around the women’s perceptions of their religion’s acceptability of contraceptive use are consistent with some earlier studies from similar contexts. For example, a recent study among religious leaders from Burkina Faso demonstrates that while the religious leaders (of varying religions) had high knowledge of family planning, there was generally low acceptability of its use (Barro & Bado, 2021; Barro et al., 2021). This is consistent with our finding that women from Burkina Faso generally perceived that their religions were not supportive of family planning use. Further, a recent study from Niger demonstrated that religious leaders had mixed opinions on family planning use (PSI, 2017) which corresponds to our findings that among the young women in Niger, there was perceived acceptability of use among several Muslim and Christian youth as well as cases where use was not perceived as acceptable.

Our study is also consistent with earlier studies of women of all ages that examined qualitatively the role of religion on family planning use. For example, Abdi et al. (2020) demonstrate in two Muslim communities in Kenya (including Wajir), that women had mixed perspectives on their religion’s acceptability of family planning use; some thought it was prohibited while others thought that spacing was permitted. That said, among the women who felt that their religion did not approve of FP use, they typically justified their use based on the current economic situation (Abdi et al., 2020). Further, in a qualitative study from Tanzania (Sundararajan et al., 2019), the authors demonstrate two types of beliefs: the belief that family planning is not acceptable in the Christian or Muslim faith and people should have as many children as God has allocated; and that one has a moral responsibility to care for and protect one’s family and therefore FP use is acceptable. These findings are similar to those found here for the young, married users who may consider family planning not acceptable in their religion but were using based on their personal realities of life and their sense of personal responsibility. Use among the unmarried young women was for similar reasons but under even less accepting circumstances. These findings demonstrate that while religion is important in all three study contexts, decisions around family planning use among the young women included were less influenced by religion.

This study is not without limitations. First, the young women included in this study were those who agreed to discuss their contraceptive use and thus may be more open and motivated to use than their peers who chose not to discuss their use in this type of setting. Second, our sample does not include those young people who may not use contraception because of the influence of their religion; this means we may be including those who are more willing to go against their religious beliefs. Third, we only collect data from two study sites in each country and thus the results cannot be generalizable beyond the specific study sample and sites. Fourth, based on discussions with the country teams, we identified some objective measures of engagement with the religion to capture religious practice or religiosity; it is possible that this did not provide a good representation of religious contraceptive users. Fifth, while we were meant to include a range of marital statuses across the countries/sites, we did this better in some sites than others and thus represent the perspectives of single users less well, particularly in Kenya. Finally, this study is based solely on qualitative data and thus not generalizable. A future study across these same three countries that collects quantitative data among adolescents and youth will help to inform the generalizability of the findings here.

This study demonstrates that young women who are practicing their religion are using contraception despite their perceptions that their religious leaders and religious community do not support their use. These young people are making decisions that are best for their own and their family (or couple) situations in terms of health and well-being. It is notable that in Niger which is the country where the overwhelming majority of the population is Muslim and in Wajir with a large Muslim population, the perceptions around contraceptive use for spacing and the health of the mother were more nuanced than in Burkina Faso and among the Christians in Kenya. While religion has been suggested as an important influence on contraceptive non-use (Akinkorah, 2020; Tigabu et al., 2018; Obasohan, 2015; Alomair et al., 2020), in some of our study contexts (e.g., Niger and Wajir), Muslim women view greater flexibility in their religious teachings and thus religion is less of a barrier to young women’s contraceptive use.

Given that religion plays an important role in the lives of many young people in the study countries, programs are needed that seek to align the religious perspectives and activities with the realities of young people who are finding themselves needing to use family planning to improve their own health and well-being. This may mean undertaking activities that bring together young people, health workers, education officers, and religious leaders. A cross-sectoral approach to undertaking activities for young people will yield better results for expanding contraceptive use. For example, schools, including religious and non-religious schools, where most young people spend time should be involved in programs that train educators to discuss contraceptive use and address the nuances around religion and contraceptive use; these programs can also address access to family planning services for young people. Likewise, programs specifically targeting religious leaders can engage champion religious leaders who can share their experiences working with young people. These programs can strengthen the religious leaders’ and other key gatekeepers’ understanding of the benefits of contraception for child spacing and the health of the mother and child and also help these people to understand that some young people will use contraception with or without their religion’s support. Over time, as more religious and community leaders are engaged in programs that share this type of information, norms and perceptions around the acceptability of contraceptive use for young women will shift and more young people will have access to and be able to use contraception to meet their desire to delay, space, or limit childbearing.

## Data Availability

These data are not publicly available in order to protect the identities of the participants involved but are available from the first author (speizer@email.unc.edu) on reasonable request that clarifies how the data will be used and provides plans for safeguarding the data in a manner that protects the participants identities.

## Footnotes

1 Note that in Kenya, one woman was in the age group 25–26 and in Niger three women were in this age group. These young women were included since they met the other eligibility criteria (practicing their religion and using a modern method).
